# LncRNAs Participate in Post-Resuscitation Myocardial Dysfunction Through the PI3K/Akt Signaling Pathway in a Rat Model of Cardiac Arrest and Cardiopulmonary Resuscitation

**DOI:** 10.3389/fphys.2021.689531

**Published:** 2021-06-14

**Authors:** Jingying Hou, Chaotao Zeng, Guanghui Zheng, Lian Liang, Longyuan Jiang, Zhengfei Yang

**Affiliations:** Sun Yat-sen Memorial Hospital, Sun Yat-sen University, Guangzhou, China

**Keywords:** cardiopulmonary resuscitation, cardiac arrest, post-resuscitation myocardial dysfunction, long non-coding RNAs, competitive endogenous RNA, PI3K/Akt signaling pathway

## Abstract

In this study, we aimed to explore the role of lncRNAs in post-resuscitation myocardial dysfunction in a rat model of CA-CPR. A rat model of CA-CPR was constructed using a VF method. Myocardial functions, including cardiac output (CO), ejection fraction (EF), and myocardial performance index (MPI), were evaluated at the baseline, and 1, 2, 3, 4, and 6 h after resuscitation. A high throughput sequencing method was used to screen the differentially expressed lncRNAs, miRNAs, and mRNAs, which were further analyzed with bioinformatics. In addition, relationships between the molecules involved in the PI3K/Akt signaling pathway were explored with ceRNA network. Compared with the sham group, EF was significantly reduced and MPI was increased at the five consecutive time points in the CA-CPR group. 68 lncRNAs were upregulated and 40 lncRNAs were downregulated in the CA-CPR group, while 30 miRNAs were downregulated and 19 miRNAs were upregulated. Moreover, mRNAs were also differentially expressed, with 676 upregulated and 588 downregulated. GO analysis suggested that genes associated with cell proliferation, cell death and programmed cell death were significantly enriched. KEGG analysis showed that the PI3K/Akt, MAPK and Ras signaling pathways were the three most-enriched pathways. Construction of a ceRNA regulatory network indicated that LOC102549506, LOC103689920, and LOC103690137 might play important roles in the regulation of the PI3K/Akt signaling pathway in the CA-CPR treated rat. Taken together, LncRNAs, including LOC102549506, LOC103689920 and LOC103690137, might participate in post-resuscitation myocardial dysfunction by functioning as ceRNAs and regulating the PI3K/Akt signaling pathway.

## Introduction

Cardiopulmonary resuscitation (CPR) after cardiac arrest (CA) can result in a variety of injuries such as severe ischemia, hypoxia and energy metabolism disorders (Sekhon et al., 2017). Restoration of spontaneous circulation (ROSC) post CPR provokes serious reperfusion injury and further impairs myocardial function, which becomes the leading cause of death after resuscitation (Ha et al., 2017). In a clinical cohort study which included 28,611 patients with non-traumatic CA, only 1,591 patients survived after CPR. High readmission and mortality rates were reported in the survived patients 3 years after hospital discharge. Cardiovascular and neurological factors were the primary cause of morbidity and mortality (Shuvy et al., 2017). In consideration of this, prevention and treatment of myocardial damage and dysfunction after CPR is the key for ameliorating survival and life quality of the patients recovering from CA.

CA-CPR-ROSC is a complicated pathophysiological process, in which myocardial ischemia-reperfusion injury (MIRI) predominantly affects its progression and prognosis (Ha et al., 2017; Shuvy et al., 2017). MIRI is entangled with the release of oxygen free radicals, intracellular calcium overload, inflammatory cascade, apoptosis and mitochondrial dysfunction (Patil et al., 2015; Qin et al., 2016; Lv et al., 2017; Tan et al., 2020). Although recent studies have demonstrated its multifaceted pathogenesis, the clinical outcomes of patients remain scarcely improved. Moreover, current treatment options are relatively limited. In view of this, it is imperative to further explore the mechanism of MIRI post CPR in order to obtain novel therapeutic targets.

Long non-coding RNAs (lncRNAs) exert a pivotal role in various processes (Hou et al., 2016, 2018). Recent data show that they can act as an orchestrator in I/R injury (Shi et al., 2019; Huang et al., 2020; Su et al., 2020). In this study, we speculated that lncRNAs might be involved in MIRI and myocardial dysfunction induced by CA-CPR. A rat model of post-resuscitation myocardial dysfunction and tissue injury was constructed. Gene sequencing, KEGG and GO analyses were applied to detect the functions of different RNAs. In addition, lncRNAs associated with the PI3K/Akt signaling pathway were predicted by lncRNAs-miRNAs-mRNAs network analysis. This work may provide theoretical and experimental basis for the development of molecular targeting therapy for myocardial dysfunction in CA-CPR.

## Materials and Methods

### Animals

Healthy male Sprague-Dawley rats (6–8 months, 450 to 550 g) were obtained from Harlan Sprague-Dawley Inc (California, United States). Animal feeding and disposal were described as previously (Wang et al., 2018a; He et al., 2020). Blood temperature was monitored through a thermocouple microprobe (9030–12-D-34; Columbus Instruments, Columbus, OH, United States). Temperature was kept within 37 ± 0.5°C by using an infrared surface heating lamps (Wang et al., 2018b; He et al., 2020). Rats were euthanized by cervical dislocation after the experiment. This study adhered to ethical standards for animal research and was approved by the Animal Care and Use Committee of the Sun Yat-sen University.

### Treatment

Sixteen rats were randomized to the sham group and the CA-CPR group, respectively. A CA-CPR rat model was constructed according to our previous published work (He et al., 2020). CA was induced with VF and untreated for 8 min, followed by 8 min of CPR. Precordial compression (PC) was performed as previously described (Wang et al., 2018b; He et al., 2020). Defibrillation was attempted with up to a 4-J counter shock after 8 min of CPR (He et al., 2020). The sham group underwent the operation without CPR. The plasma separation method was performed as previously reported (Wang et al., 2018a; He et al., 2020). The separated plasma and heart tissue were immediately frozen and stored in −80°C for further applications.

### Physiology and Biochemistry Measurements

Parameters including ejection fraction (EF), cardiac output (CO) and myocardial performance index (MPI) at different time points (baseline, 1, 2, 3, 4 and 6 h after CR-CPR) were measured and calculated as provided in our previous study (He et al., 2020). Heart rates (HR), mean arterial pressures (MAP) and ETCO2 values were continuously recorded on a personal computer-based data-acquisition system supported by Common Ocean Data Access System hardware and software (DataQ, Akron, OH, United States). For arterial blood gas analysis, parameters including PaO2 pressure, core temperature, pH and lactate were measured at different time points (baseline, 1, 2, 3, 4, and 6 h after CR-CPR) by using a conventional blood gas analyzer (Phox, Stat Profile; Nova Biomedical Corporation, Waltham, MA, United States) as previously (He et al., 2020). Serum troponin I (Tn I) and N-terminal pro-brain natriuretic peptide (NT-proBNP) were measured by Elisa kits according to the manufacturer’s instructions (Cat. No. 2010-2-US; Life Diagnostics Inc; Cat. No. MBS164802; MyBioSource, Inc) (Bausch et al., 2020; Bridges et al., 2020).

### LncRNA Sequencing

MiniBEST Universal RNA Extraction Kit (TaKaRa, Japan) was used for the extraction of total RNA in the heart tissues with or without CA-CPR treatment (Cuijpers et al., 2020). The procedures were performed following the manufacturer’s instructions. For RNA integrity analysis, the extracted total RNA was detected by agarose gel electrophoresis. The detection of purity and concentration of total RNA was performed by NanoDrop 2.0 (Thermo Fisher, United States) (1.8 < OD_260/280_ < 2.1). A Ribo-Zero^TM^ kit (Vazyme, China) was used to wipe out ribosome RNA from 3 μg of total RNA (Heimerl et al., 2020). Synthesis of double-stranded complementary DNA was conducted by a random primer method with SMARTScribe^TM^ Reverse Transcriptase (TaKaRa, Japan) after RNA fragmentation. The double-stranded cDNA was ligated to adaptors after being end-repaired and A-tailed with TruSeq RNA Library Prep Kit v2 (illumine, United States). PCR amplification was employed for the enrichment of cDNA libraries. 0.8 × beads (Beckman, United States) were applied for the purification of the PCR products. Further quantification was performed by the Q-PCR method (Lin et al., 2018). The library was sequenced on an Illumina Hiseq 2500 platform with a paired-end 150 bp strategy. The construction of the RNA-seq libraries was completed by an Illumina TruSeq RNA Library Prep Kit v2. A 2 × 150 bp paired-end sequencing strategy was also carried out on the Illumina Hiseq 2500 platform. Rat genome was utilized as the reference genome. Normalization of the data was performed by using a reads per kilobase per million mapped reads method (RPKM = total exon reads/mapped reads in millions^∗^exon length in kb) (Lin et al., 2018; Liu et al., 2018). In this study, a false discovery rate with FDR ≤ 0.001 and | log2Ratio| ≥ 1 was employed to screen the differentially expressed genes between the two groups. The heatmap was portrayed with software package in R with default settings. GO and KEGG pathway analyses were carried out by using DAVID^1^ according to the default actions (Lin et al., 2018; Liu et al., 2018).

### Interaction Analysis

In this study, Cytoscape 3.7.1^2^ was employed to predict the lncRNAs-miRNAs-mRNAs network. All the parameters were analyzed according to the software default settings (Lin et al., 2018; Liu et al., 2018).

### Statistical Analysis

SPSS for windows version 19 (Chicago, IL, United States) was used for statistical analysis. All the data were described as mean ± SD (standard deviation) and compared by using student’s *t* test. A *P* value less of 0.05 was considered to be statistically significant.

## Results

### Alterations of the Physiological and Biochemical Indexes in the CA-CPR Treated Rats

**FIGURE 1:**
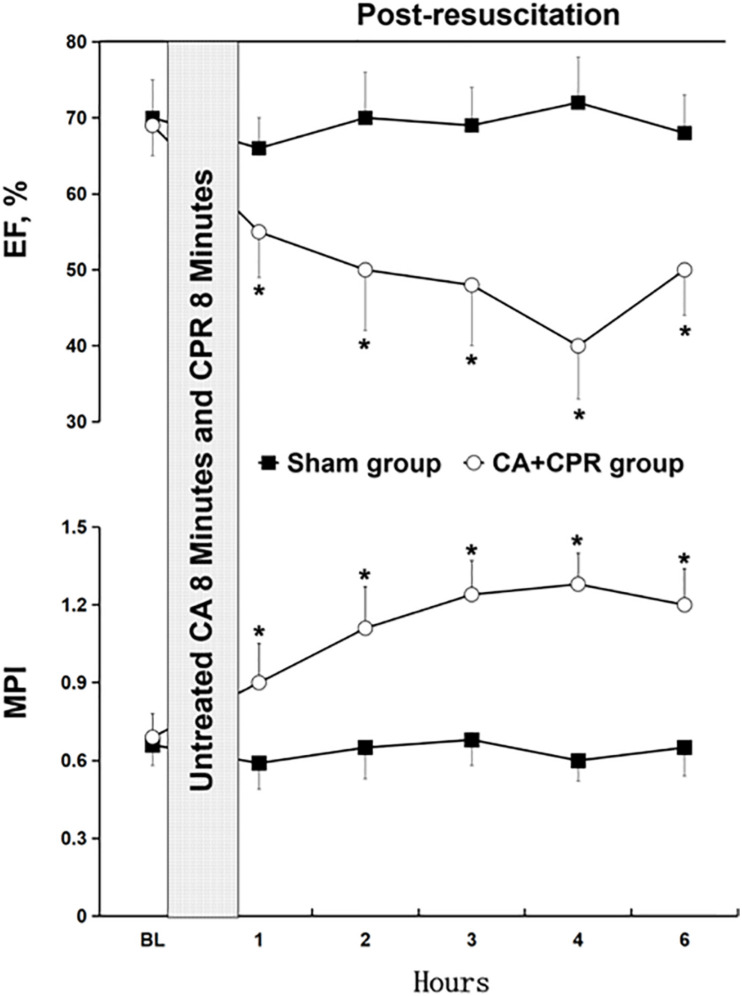
Changes of the relevant indexes of myocardial dysfunction. BL, baseline; CA, cardiac arrest; CPR, cardiopulmonary resuscitation; EF, ejection fraction; MPI, myocardial performance index. *P < 0.05 vs. the sham group.

In this study, we evaluated the relevant physiological and biochemical indexes in the two experimental groups, including CO, EF, MPI, weight, HR, MAP, PH, PaO2, lactate, ETCO2 and core temperature (Figure 1 and Table 1). No differences were observed in the parameters between the two groups at the baseline (*P* > 0.05), with the exception of EF and MPI (Table 1). Compared to the sham group, EF was significantly reduced at the five consecutive time points in the CA-CPR group, whereas MPI was obviously increased correspondingly (*P* < 0.05, *P* < 0.05; Figure 1).

**TABLE 1:** Baseline parameters of the two experimental groups.

Items	CA-CPR group (*n* = 8)	Sham group (*n* = 8)	*P* value
Weight (g)	505 ± 20	495 ± 25	*NS*
HR, beats/min	347 ± 18	356 ± 22	*NS*
MAP, mm Hg	136 ± 18	140 ± 26	*NS*
PH	7.44 ± 0.15	7.50 ± 0.12	*NS*
PaO_2_, mm Hg	93 ± 12	96 ± 17	*NS*
Lactate, mmol/L	0.7 ± 0.5	0.8 ± 0.4	*NS*
ETCO_2_, mm Hg	45 ± 4	44 ± 3	*NS*
Core Temperature,°C	36.9 ± 0.4	36.7 ± 0.5	*NS*

*HR, heart rate; MAP, mean artery pressure. Values were presented as mean ± SD. *NS*, non-significant.*

### Sequencing

**FIGURE 2:**
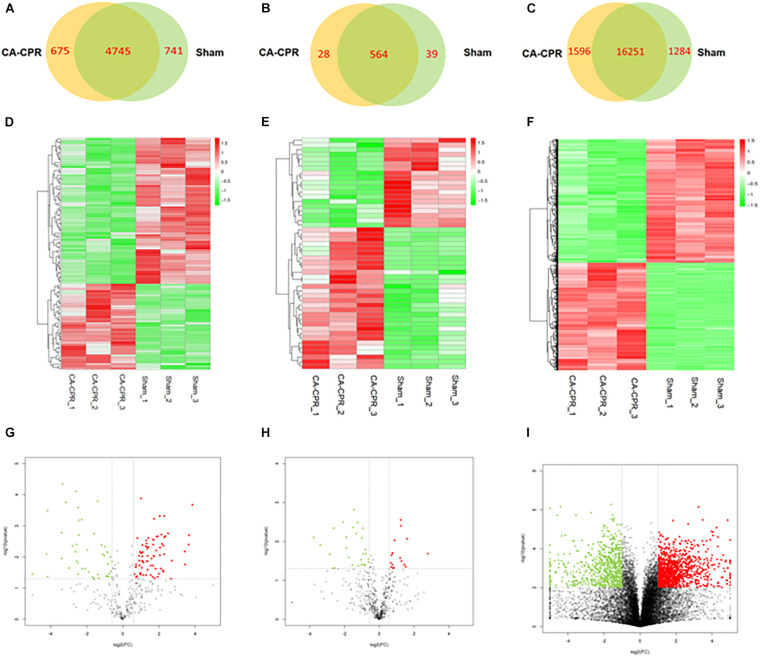
Analysis of the RNAs expression profiles in the two groups. **(A)** to **(C)** showed the Wayne diagram analysis of all the differentially expressed lncRNAs **(A)**, miRNAs **(B)** and mRNAs **(C)** in the rat heart tissues of the two groups; **(D)** to **(E)** showed the heatmap clustering analysis of the differentially expressed lncRNAs **(D)**, miRNAs **(E)**, and mRNAs **(F)** between the two groups; **(G)** to **(H)** showed the volcano atlases of the differentially expressed lncRNAs **(G)**, miRNAs **(H)** and mRNAs **(I)** in the two groups.

A high throughput sequencing was applied to explore the RNA expression profile. A 5420 lncRNAs and 5486 lncRNAs could be harvested in the CA-CPR group and the sham group respectively, (Figure 2A). The overlap between the two data sets suggested that 4645 lncRNAs could be obtained. Additionally, 108 lncRNAs were differentially expressed in the CA-CPR group, with 68 up-regulated and 40 down-regulated (Figures 2B,C). The top 20 were listed in Table 2, which included AC110709.2, AABR07042668.1 and AC133316.1. Detection of the miRNA expression profile showed that 592 miRNAs and 603 miRNAs could be obtained in the CA-CPR group and the sham group respectively, and the overlap between the two data sets contained 564 miRNAs in total (Figure 2D). Further analysis indicated that 49 miRNAs were differentially expressed in the CA-CPR group, with 19 up-regulated and 30 down-regulated (Figures 2E,F). The top 20 miRNAs of significant difference were listed in Table 3, which included miR-496-3p, miR-6328 and miR-141-5p. For the expression profile of mRNAs, 17847 mRNAs and 17535 mRNAs could be discovered in the CA-CPR and the sham group respectively. The overlap between the two data sets covered 16251 mRNAs (Figure 2G), and 1264 mRNAs were found to be differentially expressed, with 676 up-regulated and 588 down-regulated in the CA-CPR group (Figures 2H,I). The top 20 differentially expressed mRNAs were listed in Table 4, which included Lrrc10, Mmp15, and G0s2.

**TABLE 2:** The top 20 differentially expressed lncRNAs detected by high-throughput sequencing analysis.

Gene	CA-CPR_1	CA-CPR_2	CA-CPR_3	Sham_1	Sham_2	Sham_3	Fold change	*P*-value
AC110709.2	0.408655	0.044487	0.073818	2.642661	2.246608	2.687576	14.37840633	0.000209343
AABR07042668.1	0.062302	0.032022	0	0.338484	0.507278	0.330221	12.46748442	0.003963165
AC133316.1	0.227657	0	0.14414	1.05754	1.026248	1.961857	10.88132771	0.017524147
AC117065.1	0.209503	0	0	0.673176	0.608366	0.971024	10.75195105	0.006647749
LOC108349943	0.104601	0.104799	0	0.619313	0.34753	0.397594	6.515936008	0.013117983
AABR07027407.1	0.260327	0.256484	0.350918	1.61495	2.857195	0.98569	6.289792089	0.049902088
AABR07061448.2	0.187434	0.079447	0.054715	0.707573	0.596682	0.522588	5.680552619	0.001744076
AABR07007026.1	0.176197	0.27401	0.198691	1.292506	1.151826	0.874383	5.114386236	0.0021416
AABR07031489.1	1.070988	1.092567	0.521277	3.837565	4.530953	4.763746	4.891279603	0.000484377
AABR07006475.1	0.409313	0.436174	0.31449	2.483035	1.725255	1.463557	4.889620225	0.008134905
AABR07054460.4	9.86921	10.11746	11.399301	1.789125	2.130902	1.344989	0.167750617	7.79284E-05
Rc3h1	0.289336	0.36667	0.484402	0.045082	0.110856	0.0311	0.164009723	0.006785801
LOC103692471	1.883408	2.563742	2.008501	0.78971	0.108022	0.040284	0.145301535	0.004428847
AABR07065124.1	0.279885	0.550305	0.387986	0.034415	0	0.082298	0.095809637	0.011070341
AABR07015917.1	0.8805	1.035362	0.682423	0.105599	0.048009	0.094824	0.095613838	0.001643598
AABR07032261.1	1.179859	1.182772	1.47847	0	0.102583	0.111385	0.055704862	0.000328549
AABR07066529.3	5.83767	15.02352	6.555665	0.459186	0.80895	0.257205	0.055635156	0.043171904
Rn60_20_0141.4	0.702785	0.753476	0.389023	0	0	0.099107	0.053708264	0.008016014
AABR07044454.1	0.702296	2.492548	2.588051	0	0	0.057506	0.009944154	0.03586021
AABR07021357.1	0.189619	0.375452	0.344635	0	0	0	0	0.006196935

**TABLE 3:** The top 20 differentially expressed miRNAs detected by high-throughput sequencing analysis.

miRNA	CA-CPR_1	CA-CPR_2	CA-CPR_3	Sham_1	Sham_2	Sham_3	Fold change	*P*-value
miR-496-3p	0	0.142782101	0	0.446645381	0.272803881	0.266904995	6.908108593	0.02063276
miR-6328	0.316256235	0.142782101	0.598039125	1.191054348	0.954813584	1.06761998	3.03997392	0.008530964
miR-141-5p	0.158128117	0.142782101	0.23921565	0.744408968	0.409205822	0.400357493	2.877055837	0.044768706
miR-708-3p	2.846306111	1.713385208	1.196078251	7.146326089	3.955656275	4.537384916	2.71716355	0.039647754
miR-6314	1.897537408	0.999474705	1.554901726	4.168690218	4.228460156	2.402144955	2.425764676	0.031776525
miR-31b	0.790640586	0.428346302	0.71764695	1.637699729	1.636823286	1.334524975	2.379927427	0.004005507
miR-671	4.11133105	2.284513611	3.229411277	8.188498643	6.820097025	7.606792358	2.349588226	0.002789938
miR-760-3p	0.632512469	0.285564201	0.23921565	1.042172555	0.818411643	0.800714985	2.299591154	0.026681912
miR-664-2-5p	3.478818581	2.998424114	4.066666053	7.592971469	6.001685382	5.871909891	1.846238165	0.009354439
miR-490-5p	9.171430803	6.710758731	6.578430379	17.12140625	11.32136106	11.61036728	1.783260424	0.046996599
miR-19b-1-5p	0.158128117	0.142782101	0.358823475	0	0	0	0	0.034191646
miR-501-5p	0.316256235	0.142782101	0.23921565	0	0	0	0	0.009747454
miR-98-3p	0.790640586	0.428346302	0.598039125	0	0	0.133452498	0.073445562	0.007844817
miR-212-3p	1.581281173	0.856692604	1.794117376	0	0.272803881	0.133452498	0.095994241	0.012341226
miR-449a-5p	1.73940929	2.427295711	3.827450403	0.446645381	0.136401941	0.53380999	0.139709232	0.021549325
miR-223-3p	35.10444204	107.800486	118.292139	12.80383424	11.457763	17.34882468	0.15930662	0.049217025
miR-146b-3p	6.799509044	5.140155624	8.372547756	0.893290761	0.682009703	1.734882468	0.162965159	0.004559746
miR-223-5p	3.636946698	9.994747047	11.84117468	1.637699729	0.818411643	1.868334965	0.16976676	0.04812294
miR-335	0.474384352	0.571128403	0.71764695	0.148881794	0.136401941	0.133452498	0.237491947	0.003200333
miR-337-5p	0.790640586	0.999474705	1.315686076	0.297763587	0.272803881	0.266904995	0.269647786	0.007790613

**TABLE 4:** The top 20 differentially expressed mRNAs detected by high-throughput sequencing analysis.

Gene	CA-CPR_1	CA-CPR_2	CA-CPR_3	Sham_1	Sham_2	Sham_3	Fold change	*P*-value
Lrrc10	6.61346	3.388767	1.511001	90.415512	102.310379	121.696075	27.30962733	0.000397761
Mmp15	2.069283	1.073066	1.283242	20.144276	16.399656	21.377337	13.08780432	0.0003075
G0s2	3.816252	3.784888	4.3392	54.451767	43.740429	52.194508	12.59484269	0.000145437
Pik3ip1	1.193658	1.072336	1.023903	11.463047	11.619609	13.212208	11.03221894	3.99E-05
Mxra8	1.753767	1.4485	1.510603	11.864681	12.978195	15.195252	8.495487463	0.000278695
Lrrc14b	3.539419	2.519828	1.541337	20.775835	17.762569	24.149925	8.247830561	0.000685269
Tob1	4.391835	4.971542	4.178354	35.58387	34.999737	30.367887	7.454844141	6.29E-05
Ppargc1a	3.45969	2.954994	2.179827	23.277143	19.970644	19.825525	7.338790072	0.000106477
Angptl2	4.388309	1.319822	2.263174	20.388247	16.88448	20.948242	7.303819011	0.000428772
Mettl7a	5.27579	2.115927	2.126382	19.777586	22.551233	26.080688	7.187307781	0.0007364
Timp1	224.022034	289.615356	393.648682	6.966794	8.110912	9.475581	0.027062343	0.003984694
Fgl2	209.985962	334.000397	234.444839	6.244466	7.874391	8.512517	0.029073056	0.002667085
Rnd1	107.459427	154.349442	139.470978	3.92154	5.604002	3.429323	0.032283866	0.000729726
Lmcd1	737.330933	859.736145	1123.62207	32.472263	40.584961	30.783257	0.038166977	0.001568582
Hbegf	42.674644	52.960445	80.786942	2.304341	2.348645	2.324754	0.03955141	0.007697695
Thbs1	269.073212	387.639648	362.750366	11.702993	21.531965	9.550546	0.041968659	0.000853814
Myc	81.309822	102.588875	84.98732	3.479075	4.231797	3.898081	0.043174253	0.000198844
Serpine1	631.961304	628.860657	465.4487	23.035692	39.601185	13.947671	0.04436416	0.000582672
Tnfrsf12a	212.441345	222.3116	243.840561	8.930306	14.618487	9.87612	0.049256164	2.20E-05
‘Rgs16	35.719707	26.219276	47.280396	1.455736	1.83702	2.115744	0.0495196	0.004742809

### Functional Analysis

**FIGURE 3:**
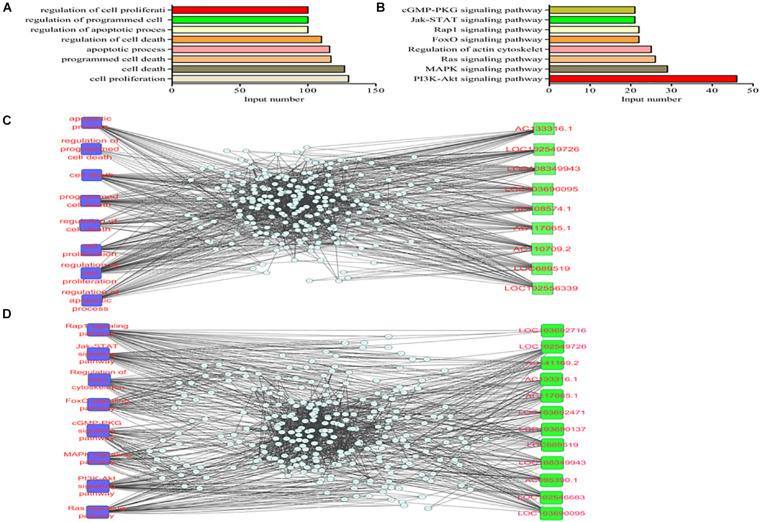
GO and KEGG analyses of the differentially expressed mRNAs in the two groups. **(A)** GO analysis; **(B)** KEGG analysis; **(C)** lncRNAs-miRNAs-mRNAs interaction network of the key genes; **(D)** lncRNAs-miRNAs-mRNAs interaction network of the key pathways.

The functional analysis was further performed based on the aforementioned data, GO terms with a corrected *P*-value of less than 10^–4^ were considered to be significantly enriched. Biological processes, cellular components and molecular functions were shown in Figure 3A. Cell proliferation, cell death and programmed cell death were the three top enriched terms. PI3K/Akt, MAPK and Ras signaling were the three most enriched pathways in the KEGG analysis (Figure 3B). Moreover, we constructed the lncRNAs-miRNAs-mRNAs interaction network with the key GO items (Figure 3C). The lncRNAs in the network included AC133316.1, LOC102549726 and LOC108349943. Meanwhile, the lncRNAs-miRNAs-mRNAs interaction network was also constructed with the key KEGG pathways (Figure 3D). The lncRNAs in this network included LOC103692716, LOC102549726 and AC141169.2.

### PI3K/Akt Signaling Pathway and the Relevant ceRNA Network

**FIGURE 4:**
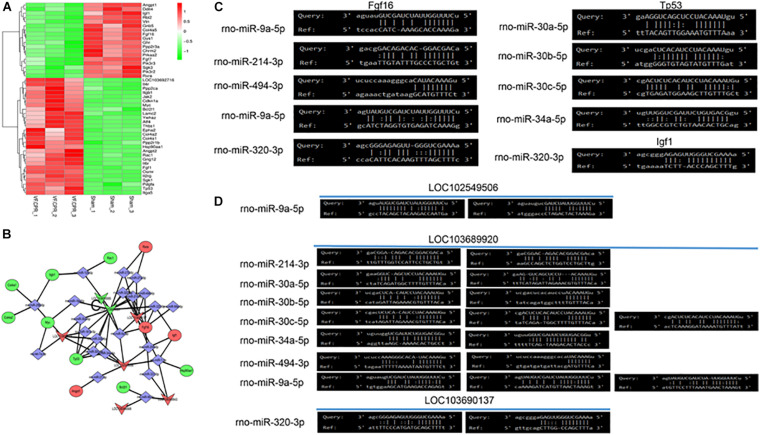
Bioinformatics analysis of the key lncRNAs involved in the PI3K/Akt signaling pathway. **(A)** Differentially expressed mRNAs in the PI3K/Akt signaling pathway post the CA-CPR treatment; **(B)** Analysis of the ceRNA effects of the lncRNAs that were involved in the PI3K/Akt signaling pathway; **(C)** Bioinformatics analysis of the miRNAs-mRNAs mutual interaction; **(D)** Bioinformatics analysis of the lncRNAs-miRNAs mutual interaction.

Figure 4A showed the heatmap of the differentially expressed genes in thePI3K/Akt signaling pathway. It was revealed that 28 genes were significantly up-regulated and 18 genes were down-regulated in the CA-CPR group compared with the sham group. A ceRNA network diagram was drawn based on the differentially expressed genes in the PI3K/Akt signaling pathway (Figure 4B). It was indicated that LOC103696137, LOC103689920, LOC102549506, LOC108348568, LOC103690095 and LOC108349943 were tightly correlated with the PI3K/Akt signaling pathway. The lncRNAs-miRNAs interaction network showed that LOC103696137, LOC103689920 and LOC102549506 could interact with various miRNAs. In addition, the miRNAs-mRNAs interaction network suggested that Fgf16, Tp5 and Igf could be regulated by multiple miRNAs (Figure 4C). This mutual interplay comprised a complex lncRNAs-miRNAs-mRNAs regulatory network in the CA-CPR rat model (Figure 4D).

## Discussion

In the present study, we successfully reproduced post-resuscitation myocardial dysfunction and tissue injury in a rat model of CA-CPR and discovered a number of differentially expressed RNAs. Furthermore, relevant signaling pathways were detected through the functional analysis and the PI3K/Akt signaling was found to be the most enriched one. The subsequent lncRNAs-miRNAs-mRNAs network analysis suggested that several specific lncRNAs, including LOC102549506, LOC103689920 and LOC103690137, might play important roles in regulating the PI3K/Akt in the rats experienced CA-CPR.

Post-CA syndromes could trigger serious organ damage or even lead to death in a large proportion of patients (Girotra et al., 2015; Neumar et al., 2015; Ha et al., 2017). Damage involved is mainly due to the tissue reperfusion injury after resuscitation, which is induced by severe hypoxia and ischemia (Eltzschig and Eckle, 2011; Shuvy et al., 2017). During the procedure of CA-CPR, a series of events affecting the body function occurs, including circulatory arrest, hypoxemia, acidosis, I/R injury, stress response and metabolic disorders (Patil et al., 2015). Although reperfusion is critical for the recovery of cardiac function, it can cause the impairment and dysfunction of myocardial cells after CPR (Angelos et al., 2011). In this study, we tried to investigate post-resuscitation myocardial dysfunction through the CA-CPR model. Relevant physiological and biochemical indexes were assessed after the model construction. It was found that EF was significantly reduced, while MPI was obviously increased at the five consecutive time points after the baseline in the CA-CPR group, indicating that both myocardial contractility and global cardiac function were impaired post-resuscitation from CA. However, the underlying molecular mechanism was still unclear. Previous investigations have been concentrated on the pathogenesis of myocardial dysfunction caused by global MIRI (Kalogeris et al., 2016; Zhou et al., 2018, 2020; Heusch, 2019; Wang et al., 2020a,b). In spite of this, signaling molecules dominating post-resuscitation myocardial dysfunction remain elusive and need to be further explored.

Non-coding RNAs (ncRNAs), including lncRNAs and miRNAs, exert vital functions in the regulation of proteins. These molecules participate in various organism processes (Li et al., 2019a; Jusic et al., 2020). They also modulate numerous diseases, including diseases of the nervous and cardiovascular systems (ES et al., 2018; Paulin et al., 2020). The regulatory role of lncRNAs has already been established in previous researches (ES et al., 2018; García-Padilla et al., 2018). Additionally, lncRNAs are crucial mediators in the I/R injury of several organs, including the heart (Zeng et al., 2019; Liang et al., 2020). Microarray analysis has been used in a mouse model to investigate the differential expression profile of lncRNAs in the initial stage of reperfusion following myocardial ischemia (Liu et al., 2014). Another study focusing on cerebral lncRNA and mRNA expression profiles post-resuscitation from CA has already verified the lncRNAs-mRNAs interaction network. It is revealed that the interplay between lncRNAs and mRNAs mediates the key metabolic pathways and processes in the rat cerebral cortex in CA-ROSC (Liu et al., 2018). In addition to lncRNAs and mRNAs, miRNAs has also drawn considerable attention in recent years (Forini et al., 2014; Wang et al., 2020c). Substantial studies have sustained their potential in the therapy of MIRI (Shao et al., 2018; Wu et al., 2019; Wang et al., 2020c). Previous functional studies of lncRNAs in MIRI are mostly based on the acute myocardial infarction (AMI) model (Liu et al., 2014). However, the expression profiles of different RNAs and their mutual regulatory networks in post-resuscitation myocardial dysfunction have not been reported. Here, we first synchronously detected the lncRNAs, miRNAs and mRNAs expression profiles and their regulatory networks in a rat model of CA-CPR. High-throughput sequencing analysis was applied for the procedure. Differentially expressed lncRNAs, miRNAs and mRNAs were discovered in the heart tissues obtained from a CA-CPR rat. In the 108 lncRNAs transcripts, 68 were up-regulated and 40 were down-regulated. Analysis of the miRNAs showed that 49 miRNAs were differentially expressed, with 19 up-regulated and 30 down-regulated. The relevant mRNA expression profile also revealed 1264 differentially expressed gene transcripts, in which 676 were up-regulated and 588 were down-regulated. These indicated post-resuscitation myocardial dysfunction could be regulated by multiple gene levels, including diverse ncRNAs and mRNAs.

GO and KEGG analyses were further performed. It was suggested that the PI3K/Akt, MAPK and Ras signaling pathways were the three most enriched pathways, in which the PI3K/Akt signaling was on the top in this study. PI3K/Akt executes important functions in multifarious biological processes (Yudushkin, 2019). Its roles in MIRI has been recorded, and it can affect the outcome of cardiomyocytes undergoing I/R treatment (Chi et al., 2019; Liang et al., 2019). Currently, apoptosis and autophagy have been recognized as the predominant regulatory mechanisms in the pathophysiological process of MIRI (Del Re et al., 2019; Hughes et al., 2020; Wang et al., 2020b). Apoptosis and autophagy can be detected in both acute and chronic ischemia of the myocardium (Zhang et al., 2020). PI3K/Akt is one of the classic signaling transduction pathways that are involved in apoptosis and autophagy (Heras-Sandoval et al., 2014). Recent evidence reveals that PI3K/Akt can function as a downstream signaling of lncRNAs in the pathogenesis of MIRI (Li et al., 2019b; Sun et al., 2019). It has been demonstrated that lncRNAs can deteriorate autophagy and apoptosis in MIRI by targeting PI3K/Akt (Li et al., 2019b; Sun et al., 2019).

LncRNAs also play a role as ceRNAs in the process of MIRI. There are already some reports concerning the regulatory role of lncRNAs as ceRNAs in the models of MIRI. It is discovered that lncRNA AK139328 can obstruct cardiomyocyte autophagy to further mitigate MIRI by suppressing the expression of miR-204-3p (Yu et al., 2018). LncRNA-TUG1 modulates the progression of I/R injury after AMI. It exacerbates the ischemic myocardial injury by sponging miR-132-3p, which further activates HDAC3 (Daiber and Münzel, 2020; Su et al., 2020). Inhibition of this lncRNA may block MIRI following AMI (Shi et al., 2019). Another study exhibits that knockdown of lncRNA Gpr19 decreases oxidative stress and apoptosis in MIRI after AMI by regulating miR-324-5p and Mtfr1 (Huang et al., 2020). It has been pointed out that lncRNAs can act as ceRNAs in I/R injury by activating PI3K/Akt (Tian et al., 2019). LINC00520 drives the progression of renal I/R injury through the activation of PI3K/Akt. One recent study has found that lncRNA RMRP aggravates apoptosis of cardiomyocytes in MIRI through inhibiting the miR-206/ATG3 axis (Kong et al., 2019; de Jel et al., 2020; Deckelbaum et al., 2020). Further investigations in the same study show that overexpression of RMRP activates the PI3K/Akt/mTOR pathway, whereas upregulation of miR-206 can reverse the effects caused by RMRP (Kong et al., 2019).

In the present work, distinct differentially expressed lncRNAs in the PI3K/Akt signaling were further explored. The downstream mechanism was analyzed with the construction of the ceRNA network afterward. We speculated that the upregulated lncRNAs, including LOC103696137, LOC103689920, LOC102549506, LOC108348568, LOC103690095 and LOC108349943 could evoke the activation of PI3K/Akt signaling and further result in myocardial dysfunction post I/R injury in the CA-CPR rat. The binding sites of miRNAs-mRNAs and lncRNAs-miRNAs were predicted through the computer software. The following loops were ultimately identified as the functional ceRNA networks: LOC102549506/miR-9a-5p/Fgf16, LOC103689920/miR-214-3p/Fgf16, LOC103689920/miR-30a-5p/Tp53, LOC103689920/miR-30b-5p/Tp53, LOC103689920/miR-30c-5p/Tp53, LOC103689920/miR-34a-5p/Tp53, LOC103689920/miR-494-3p/Fgf16, LOC103689920/miR-9a-5p/Fgf16, LOC103696137/miR-320-3p/Igf1 and LOC103696137/miR-320-3p/Tp53. Some of the miRNAs in these loops are implicated in MIRI, such as miR-30a, miR-30c, miR-34a and miR-494-3p. Downregulation of miR-30a is correlated with impaired mitochondrial dysfunction and aggravation of cell apoptosis and necrosis in MIRI (Forini et al., 2014). The protective role of miR-30c-5p in the pathogenesis and treatment of MIRI has already been validated. Overexpression of miR-30c-5p potentiates cell viability and attenuates cardiomyocyte damage and apoptosis after MIRI (Zhou et al., 2019; Wang et al., 2020c). miR-34a is involved in autophagy. It can repress autophagy after MIRI by targeting TNF-α (Shao et al., 2018). miR-494-3p is capable of resisting sepsis-provoked myocardial injury and preserving the function of cardiomyocytes (Wu et al., 2019; Zhu et al., 2020). Taken together, relevant molecules in the ceRNA loops might serve as novel targets for treating post-resuscitation myocardial dysfunction.

In summary, a successful post-resuscitation myocardial dysfunction model was constructed in this study. A high-through put sequencing method revealed masses of differentially expressed lncRNAs, miRNAs and mRNAs. Functional analysis indicated that PI3K/Akt was the most enriched signaling pathway. Interaction network analysis of lncRNAs-miRNAs-mRNAs suggested that LOC102549506, LOC103689920 and LOC103690137 might play important roles in the regulation of the PI3K/Akt signaling pathway. This work has provided a clue for the illumination of the mechanisms underlying post-resuscitation myocardial dysfunction. It will also be conducive for the development of new therapeutic strategies to treat post-resuscitation myocardial dysfunction based on targeting the PI3K/Akt signaling pathway and relevant ceRNA networks.

However, there were some limitations in the current study. We completed only the initial sequence screening in the rat model of CA-CPR, further validation of the functions and mechanisms of the target lncRNAs need to be conducted. Get or loss of the functions of these screened molecules will be performed in future studies in order to uncover the versatile roles of these molecules.

## Data Availability Statement

The data generated in this study can be found in SRA using the accession https://www.ncbi.nlm.nih.gov/sra/PRJNA722823.

## Ethics Statement

The animal study was reviewed and approved by the Animal Care and Use Committee of the Sun Yat-sen University.

## Author Contributions

JH and CZ: bioinformatics analysis and writing of the manuscript. GZ: animal model construction and statistical analysis. LL: physiology and biochemistry measurements. ZY and LJ: study design. All authors read and approved the final manuscript.

## Conflict of Interest

The authors declare that the research was conducted in the absence of any commercial or financial relationships that could be construed as a potential conflict of interest.

## Footnotes

1
https://david.ncifcrf.gov/

2
https://cytoscape.org/
